# *N*-(4-Amino-1,2,5-oxa­diazol-3-yl)formamide

**DOI:** 10.1107/S2414314625007205

**Published:** 2025-08-15

**Authors:** Firudin I. Guseinov, Tuncer Hökelek, Elena V. Shuvalova, Aida I. Samigullina, Nizami A. Ekberov, Khudayar I. Hasanov, Mohammed Hadi Al-Douh

**Affiliations:** a Kosygin State University of Russia, 117997 Moscow, Russian Federation; b N. D. Zelinsky Institute of Organic Chemistry, Russian Academy of Sciences, 119991 Moscow, Russian Federation; c Hacettepe University, Department of Physics, 06800 Beytepe-Ankara, Türkiye; d Azerbaijan State Pedagogical University, 68 Uzeyir Hajibeyov St., AZ 1000, Baku, Azerbaijan; e Azerbaijan Medical University, Scientific Research Centre (SRC), A. Kasumzade St. 14, AZ1022 Baku, Azerbaijan; f Chemistry Department, Faculty of Science, Hadhramout University, Mukalla, Hadhramout, Yemen; Vienna University of Technology, Austria

**Keywords:** crystal structure, non-covalent inter­actions, 2,2-dihalo-3-oxoaldehydes, pyrazol

## Abstract

The asymmetric unit of the title compound, C_3_H_4_N_4_O_2_, contains two coplanar mol­ecules (*A* and *B*) completely located on mirror planes. In the crystal, N—H⋯O, N—H⋯N, C—H⋯O and C—H⋯N hydrogen bonds link the mol­ecules into sheets parallel to (010). There are neither significant π–π nor C—H⋯π(ring) inter­actions. Hirshfeld surface analysis indicates that the most important contributions to the crystal packings of mol­ecules *A* and *B* are from H⋯O/O⋯H (32.4% for *A*, 30.1% for *B*), H⋯N/N⋯H (28.2%, 31.5%) and H⋯H (12.3%, 8.0%) inter­actions.

## Abstract 2

Two crystallographically independent planar mol­ecules are present in the title compound that differ slightly in some of the bond angles.

## Graphical abstract

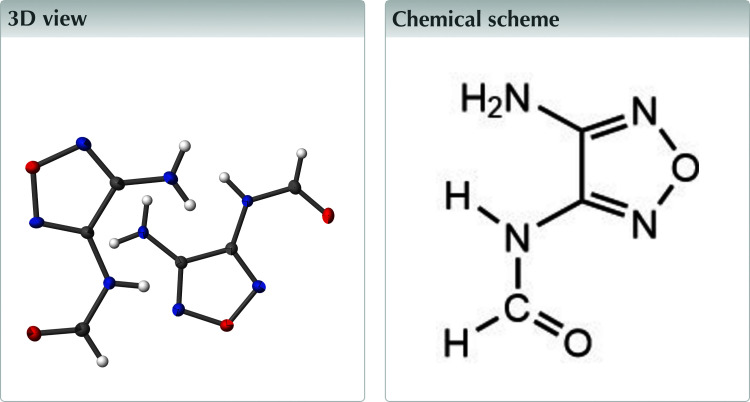

## Structure description

Oxa­diazole is a five-membered heterocyclic compound with one oxygen and two nitro­gen atoms. The oxa­diazole scaffold is a commonly utilized pharmacophore and has been subjected to extensive studies in recent years because of its metabolic profile and ability to engage in hydrogen-bonding with receptor sites (Khan *et al.*, 2017 ▸; Khalilov, 2021 ▸). Oxa­diazole derivatives have also attracted significant attention because of their reactivity (Guseinov *et al.*, 2024 ▸), diverse functional (Aliyeva *et al.*, 2024 ▸) and pharmacological properties, including anti-inflammatory, anti­bacterial, anti­hypertension, muscle relaxing and anti­cancer activities (Boström *et al.*, 2012 ▸). Moreover, derivatization of the oxa­diazole synthon with non-covalent donor or acceptor sites for hydrogen-bonding inter­actions can be applied as a synthetic strategy in the improvement of functional properties of its metal complexes (Mahmudov *et al.*, 2022 ▸). Herein, we report synthesis, mol­ecular and crystal structures together with Hirshfeld surface analysis of a new aldehyde and NH-functionalized oxa­diazole derivative, C_3_H_4_N_4_O_2_.

**Figure 1:**
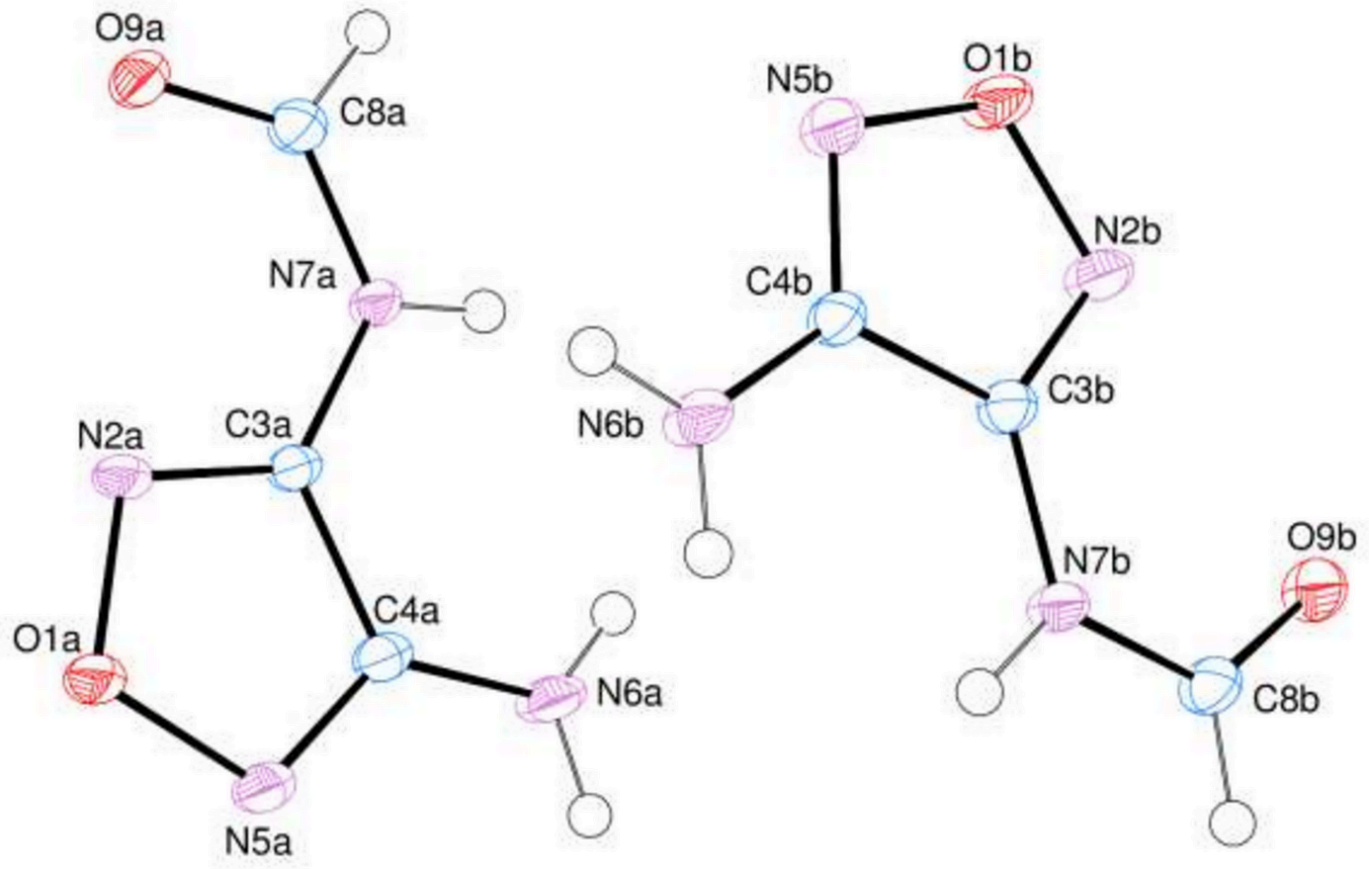
The asymmetric unit of the title compound with the atom-numbering scheme and 50% probability ellipsoids.

**Figure 2:**
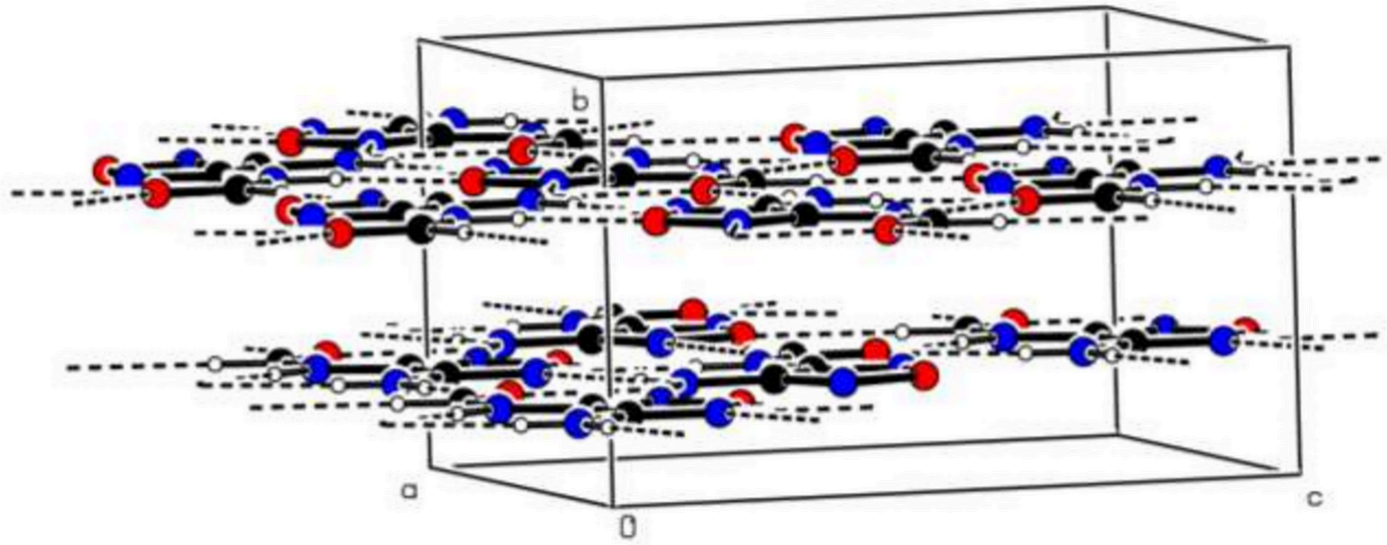
Partial packing diagram of the title compound, highlighting the layered arrangement. Inter­molecular N—H⋯N, N—H⋯O, C—H⋯O and C—H⋯N hydrogen bonds are shown as dashed lines.

The asymmetric unit contains two mol­ecules (*A* and *B*, Fig. 1 ▸) completely located on mirror planes, making the mol­ecules exactly planar. Small variations are observed in the C8*A*—N7*A*—C3*A* [125.88 (14)°] and C8*B*—N7*B*—C3*B* [125.04 (14)°], N2*A*—C3*A*—N7*A* [125.07 (14)°] and N2*B*—C3*B*—N7*B* [125.41 (14)°], N7*A*—C3*A*—C4*A* [124.71 (14)°] and N7*B*—C3*B*—C4*B* [124.10 (14)°], N5*A*—C4*A*—N6*A* [124.68 (15)°] and N5*B*—C4*B*—N6*B* [125.28 (15)°], N6*A*—C4*A*—C3*A* [127.25 (15)°] and N6*B*—C4*B*—C3*B* [126.16 (15)°], O9*A*—C8*A*—N7*A* [125.23 (15)°] and O9B—C8B—N7B [123.42 (15)°] bond angles due to the strengths of the N—H⋯O and N—H⋯N hydrogen-bonding inter­actions (Fig. 2 ▸,Table 1 ▸). Next to these classical hydrogen-bonding inter­actions, weaker C—H⋯O and C—H⋯N inter­actions are also present (Table 1 ▸), linking the mol­ecules into sheets extending parallel to (010). There are neither significant π–π nor C—H⋯π(ring) inter­actions present between mol­ecules.

**Figure 3:**
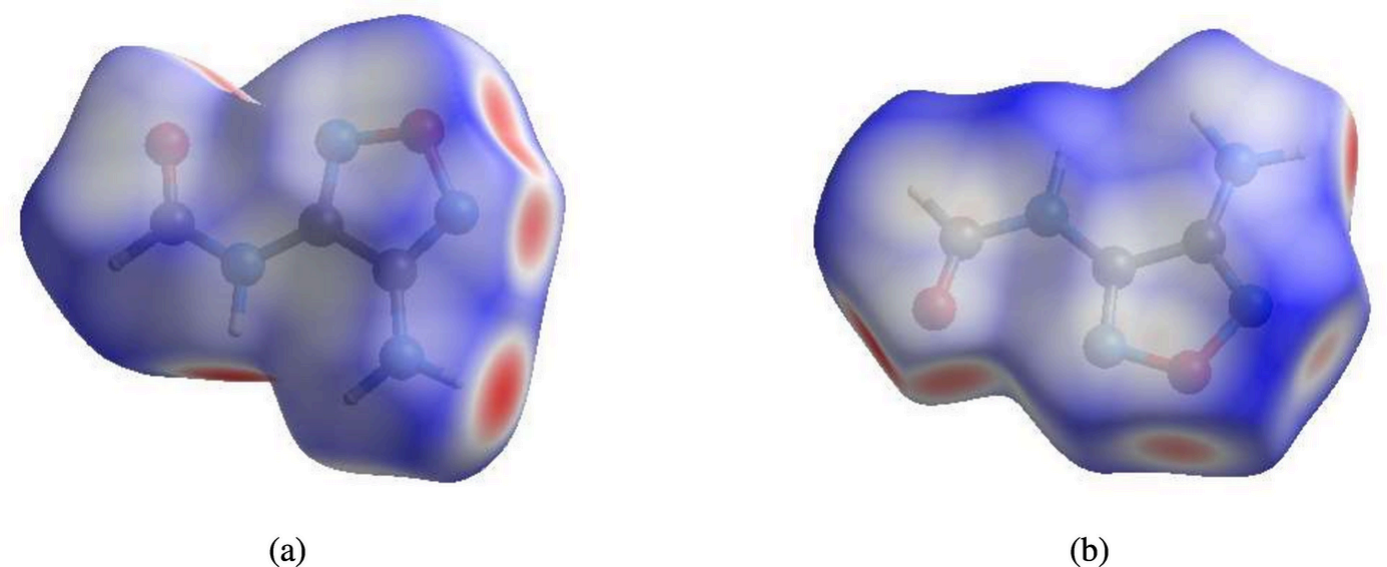
Views of the three-dimensional Hirshfeld surfaces for (*a*) mol­ecule *A* and (*b*) mol­ecule *B* plotted over *d*_norm_.

**Figure 4:**
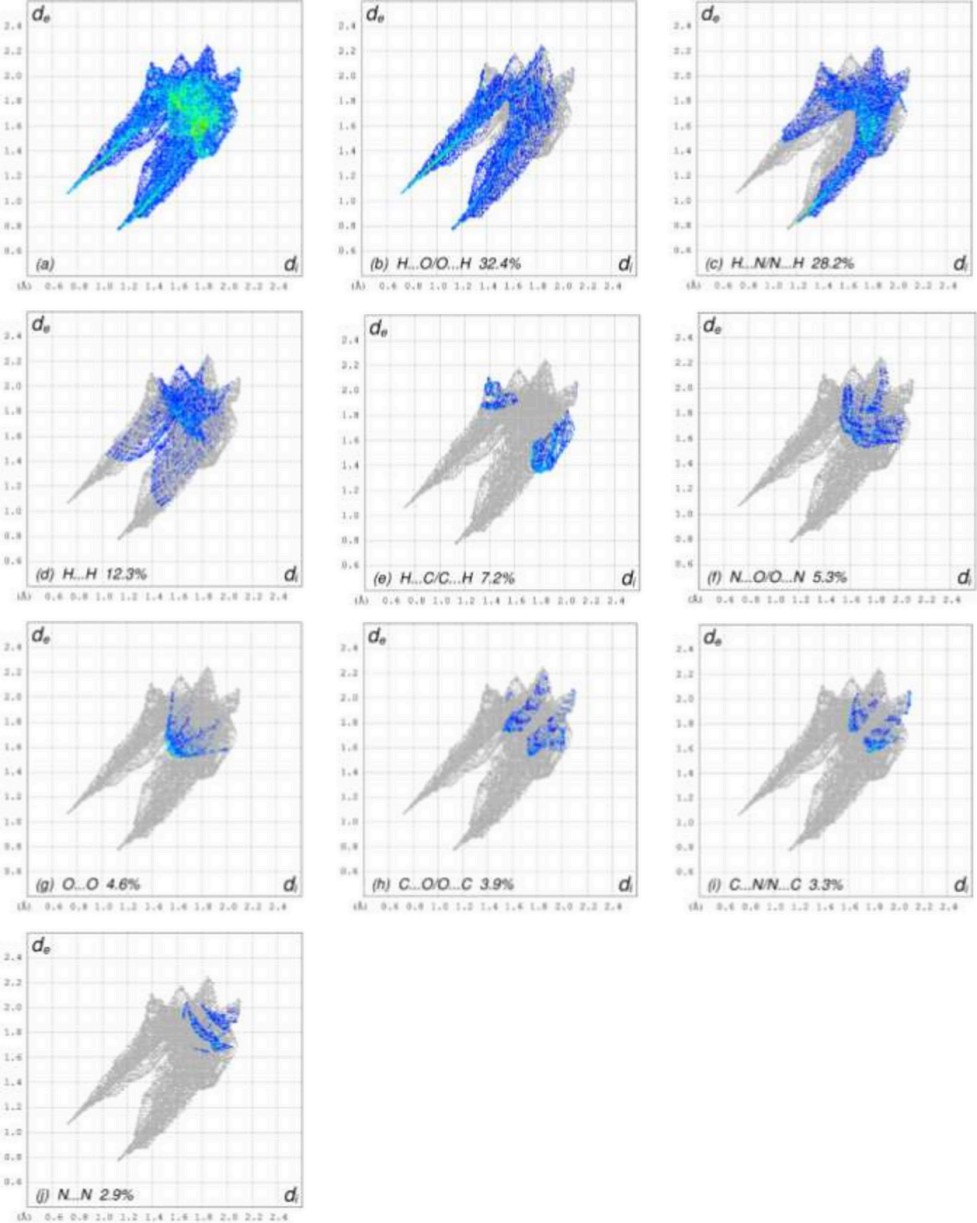
The full two-dimensional fingerprint plots for mol­ecule *A*, showing (*a*) all inter­actions, and delineated into (*b*) H⋯O/O⋯H, (*c*) H⋯N/N⋯H, (*d*) H⋯H, (*e*) H⋯C/C⋯H, (*f*) N⋯O/O⋯N, (*g*) O⋯O, (*h*) C⋯O/O⋯C, (i) C⋯N/N⋯C and (*j*) N⋯N inter­actions. The *d*_i_ and *d*_e_ values are the closest inter­nal and external distances (in Å) from given points on the Hirshfeld surface contacts.

A Hirshfeld surface (HS) analysis was carried out using *CrystalExplorer* (Spackman *et al.*, 2021 ▸) to visualize and qu­antify the inter­molecular inter­actions. In the HSs plotted over *d*_norm_ (Fig. 3 ▸*a,b*), the contact distances equal, shorter and longer with respect to the sum of van der Waals radii are shown by white, red and blue colours, respectively. According to the two-dimensional fingerprint plots, H⋯O/O⋯H, H⋯N/N⋯H and H⋯H contacts make the most important contributions to the HSs (Table 2 ▸, Figs. 4 ▸ and 5 ▸), and they have significant differences due to the different numbers and values of the close contacts.

## Synthesis and crystallization

A mixture of di­amino­furazan (20 mg, 0.2 mmol) and 2,2-di­chloro-3-oxo-3-phenyl­propanal (42.4 mg, 0.2 mmol) in 15 ml of CCl_4_ (dry) was boiled for 30 min. The reaction mixture was then cooled to room temperature, the precipitate filtered and recrystallized from chloro­form solution. Yield 15.4 mg (62%), ^1^H NMR (300 MHz, DMSO-*d*_6_): 10.40 (1*H*, NH), 8.75 (1*H*, CHO), 6.11 (2*H*, NH_2_). ^13^C NMR (200 MHz, DMSO-d_6_): 143.89, 147.78, 165.90.

## Refinement

Crystal data, data collection and structure refinement details are summarized in Table 3 ▸.

## Supplementary Material

Crystal structure: contains datablock(s) I. DOI: 10.1107/S2414314625007205/wm4232sup1.cif

Structure factors: contains datablock(s) I. DOI: 10.1107/S2414314625007205/wm4232Isup2.hkl

CCDC reference: 2480423

Additional supporting information:  crystallographic information; 3D view; checkCIF report

## Figures and Tables

**Figure 5:**
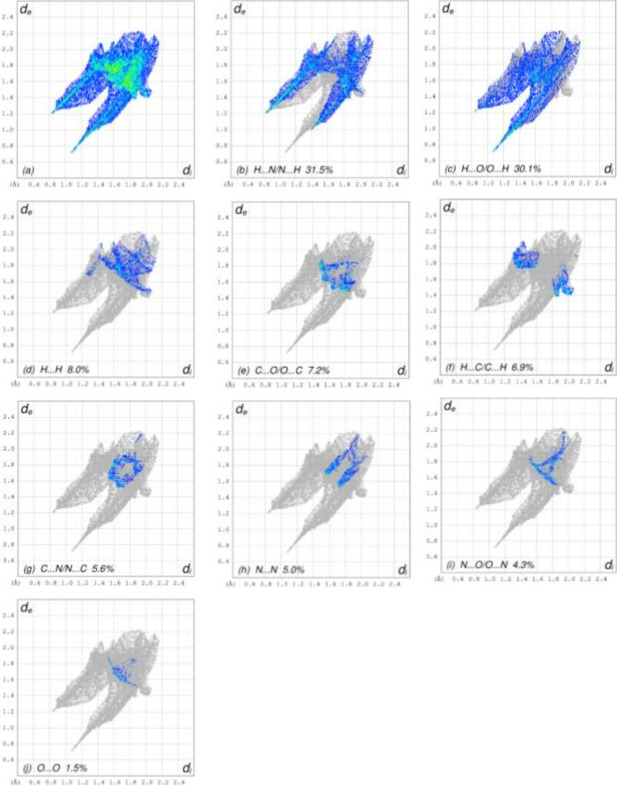
The full two-dimensional fingerprint plots for mol­ecule *B*; subdivisions are the same as in Fig. 4 ▸.

**Table 1:** Hydrogen-bond geometry (Å, °)

*D*—H⋯*A*	*D*—H	H⋯*A*	*D*⋯*A*	*D*—H⋯*A*
N6*A*—H6*A*⋯O9*A*^i^	0.88 (3)	2.05 (3)	2.9207 (19)	172 (2)
N6*A*—H6*B*⋯O9*B*^ii^	0.83 (3)	2.26 (3)	3.074 (2)	164 (2)
N7*A*—H7*A*⋯O9*B*^iii^	0.87 (3)	1.93 (3)	2.8033 (18)	177 (2)
N7*A*—H7*A*⋯O9*B*^ii^	0.87 (3)	1.93 (3)	2.8033 (18)	177 (2)
N6*B*—H6*C*⋯N5*A*^iv^	0.88 (2)	2.31 (2)	3.189 (2)	175 (2)
N6*B*—H6*D*⋯O9*A*^v^	0.85 (3)	2.31 (3)	2.8121 (19)	118 (2)
N6*B*—H6*D*⋯N2*A*^v^	0.85 (3)	2.20 (3)	3.0387 (19)	166 (3)
N7*B*—H7*B*⋯O1*A*^iv^	0.91 (3)	2.24 (3)	3.0354 (17)	145 (2)
N7*B*—H7*B*⋯N5*A*^iv^	0.91 (3)	2.32 (3)	3.2302 (19)	179 (2)
C8*A*—H8*A*⋯O1*B*^vi^	0.95	2.32	3.266 (2)	175
C8*B*—H8*B*⋯N5*B*^vii^	0.95	2.55	3.489 (2)	170

Symmetry codes: (i) 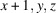; (ii) 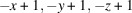; (iii) 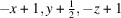; (iv) 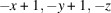; (v) 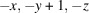; (vi) 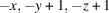; (vii) 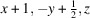.

**Table 2:** Comparison of the percentage contributions to the crystal packing for mol­ecules *A* and *B*

Contacts	*A*	*B*
H⋯O/O⋯H	32.4	30.1
H⋯N/N⋯H	28.2	31.5
H⋯H	12.3	8.0
H⋯C/C⋯H	7.2	6.9
N⋯O/O⋯N	5.3	4.3
O⋯O	4.6	1.5
C⋯O/O⋯C	3.9	7.2
C⋯N/N⋯C	3.3	5.6
N⋯N	2.9	5.0

**Table 3:** Experimental details

Crystal data	
Chemical formula	C_3_H_4_N_4_O_2_
*M*_r_	128.10
Crystal system, space group	Monoclinic, *P*12_1_/*m*1
Temperature (K)	100
*a*, *b*, *c* (Å)	7.98085 (8), 6.17409 (7), 10.19204 (9)
β (°)	95.2595 (9)
*V* (Å^3^)	500.09 (1)
*Z*	4
Radiation type	Cu *K*α
μ (mm^−1^)	1.26
Crystal size (mm)	0.28 × 0.22 × 0.04

Data collection	
Diffractometer	XtaLAB Synergy, Dualflex, HyPix
Absorption correction	Gaussian (*CrysAlis PRO*; Rigaku OD, 2023 ▸)
*T*_min_, *T*_max_	0.503, 1.000
No. of measured, independent and observed [*I* > 2σ(*I*)] reflections	13311, 1182, 1153
*R*_int_	0.031
(sin θ/λ)_max_ (Å^−1^)	0.638

Refinement	
*R*[*F*^2^ > 2σ(*F*^2^)], *wR*(*F*^2^), *S*	0.035, 0.095, 1.06
No. of reflections	1182
No. of parameters	128
H-atom treatment	H atoms treated by a mixture of independent and constrained refinement
Δρ_max_, Δρ_min_ (e Å^−3^)	0.45, −0.26

Computer programs: *CrysAlis PRO* (Rigaku OD, 2023 ▸), *SHELXT* (Sheldrick, 2015*a* ▸), *SHELXL* (Sheldrick, 2015*b* ▸), *OLEX2* (Dolomanov *et al.*, 2009 ▸) and *publCIF* (Westrip, 2010 ▸).

## full crystallographic data

### N-(4-Amino-1,2,5-oxadiazol-3-yl)formamide Crystal data

**Table:**

C_3_H_4_N_4_O_2_	*F*(000) = 264
*M**_r_* = 128.10	*D*_x_ = 1.701 Mg m^−^^3^
Monoclinic, *P*12_1_/*m*1	Cu *K*α radiation, λ = 1.54184 Å
*a* = 7.98085 (8) Å	Cell parameters from 9316 reflections
*b* = 6.17409 (7) Å	θ = 4.4–78.3°
*c* = 10.19204 (9) Å	µ = 1.26 mm^−^^1^
β = 95.2595 (9)°	*T* = 100 K
*V* = 500.09 (1) Å^3^	Prism, colorless
*Z* = 4	0.28 × 0.22 × 0.04 mm

### N-(4-Amino-1,2,5-oxadiazol-3-yl)formamide Data collection

**Table:**

XtaLAB Synergy, Dualflex, HyPix diffractometer	1182 independent reflections
Radiation source: micro-focus sealed X-ray tube, PhotonJet (Cu) X-ray Source	1153 reflections with *I* > 2σ(*I*)
Mirror monochromator	*R*_int_ = 0.031
Detector resolution: 10.0000 pixels mm^-1^	θ_max_ = 79.7°, θ_min_ = 4.4°
ω scans	*h* = −10→10
Absorption correction: gaussian (CrysAlisPro; Rigaku OD, 2023)	*k* = −7→7
*T*_min_ = 0.503, *T*_max_ = 1.000	*l* = −12→12
13311 measured reflections	

### N-(4-Amino-1,2,5-oxadiazol-3-yl)formamide Refinement

**Table:**

Refinement on *F*^2^	Secondary atom site location: difference Fourier map
Least-squares matrix: full	Hydrogen site location: mixed
*R*[*F*^2^ > 2σ(*F*^2^)] = 0.035	H atoms treated by a mixture of independent and constrained refinement
*wR*(*F*^2^) = 0.095	*w* = 1/[σ^2^(*F*_o_^2^) + (0.0568*P*)^2^ + 0.2125*P*] where *P* = (*F*_o_^2^ + 2*F*_c_^2^)/3
*S* = 1.06	(Δ/σ)_max_ < 0.001
1182 reflections	Δρ_max_ = 0.45 e Å^−^^3^
128 parameters	Δρ_min_ = −0.26 e Å^−^^3^
0 restraints	Extinction correction: SHELXL-2018/3 (Sheldrick 2015b), Fc^*^=kFc[1+0.001xFc^2^λ^3^/sin(2θ)]^-1/4^
Primary atom site location: structure-invariant direct methods	Extinction coefficient: 0.0043 (10)

### N-(4-Amino-1,2,5-oxadiazol-3-yl)formamide Special details

**Table:**

Geometry. All esds (except the esd in the dihedral angle between two l.s. planes) are estimated using the full covariance matrix. The cell esds are taken into account individually in the estimation of esds in distances, angles and torsion angles; correlations between esds in cell parameters are only used when they are defined by crystal symmetry. An approximate (isotropic) treatment of cell esds is used for estimating esds involving l.s. planes.
Refinement. The NH hydrogen atomss were located in difference-Fourier maps and were refined isotropically.

### N-(4-Amino-1,2,5-oxadiazol-3-yl)formamide Fractional atomic coordinates and isotropic or equivalent isotropic displacement parameters (Å^2^)

**Table:**

	*x*	*y*	*z*	*U*_iso_*/*U*_eq_	
O1A	0.21691 (14)	0.750000	−0.13259 (11)	0.0213 (3)	
O9A	−0.19240 (15)	0.750000	0.09542 (12)	0.0223 (3)	
N2A	0.08834 (16)	0.750000	−0.04954 (13)	0.0183 (3)	
N5A	0.37506 (17)	0.750000	−0.05892 (14)	0.0212 (3)	
N6A	0.46198 (18)	0.750000	0.16804 (16)	0.0309 (4)	
H6A	0.569 (4)	0.750000	0.154 (3)	0.034 (6)*	
H6B	0.433 (3)	0.750000	0.245 (3)	0.029 (6)*	
N7A	0.08165 (16)	0.750000	0.18320 (13)	0.0181 (3)	
H7A	0.141 (3)	0.750000	0.259 (3)	0.027 (6)*	
C3A	0.16276 (19)	0.750000	0.06877 (15)	0.0163 (3)	
C4A	0.34342 (19)	0.750000	0.06452 (16)	0.0191 (3)	
C8A	−0.0871 (2)	0.750000	0.18991 (16)	0.0205 (4)	
H8A	−0.126486	0.750000	0.275080	0.025*	
O1B	0.19712 (15)	0.250000	0.50920 (12)	0.0247 (3)	
O9B	0.71609 (15)	0.250000	0.57695 (12)	0.0226 (3)	
N2B	0.37256 (17)	0.250000	0.52444 (14)	0.0230 (3)	
N5B	0.13570 (18)	0.250000	0.37538 (14)	0.0213 (3)	
N6B	0.26997 (19)	0.250000	0.17935 (14)	0.0259 (4)	
H6C	0.365 (3)	0.250000	0.141 (2)	0.023 (5)*	
H6D	0.177 (4)	0.250000	0.130 (3)	0.039 (7)*	
N7B	0.58111 (16)	0.250000	0.37086 (13)	0.0187 (3)	
H7B	0.595 (3)	0.250000	0.283 (3)	0.028 (6)*	
C3B	0.4166 (2)	0.250000	0.40507 (15)	0.0176 (3)	
C4B	0.26997 (19)	0.250000	0.31096 (16)	0.0170 (3)	
C8B	0.7209 (2)	0.250000	0.45783 (16)	0.0201 (4)	
H8B	0.827870	0.250000	0.423922	0.024*	

### N-(4-Amino-1,2,5-oxadiazol-3-yl)formamide Atomic displacement parameters (Å^2^)

**Table:**

	*U*^11^	*U*^22^	*U*^33^	*U*^12^	*U*^13^	*U*^23^
O1A	0.0133 (5)	0.0377 (7)	0.0134 (5)	0.000	0.0029 (4)	0.000
O9A	0.0136 (5)	0.0363 (7)	0.0165 (6)	0.000	0.0000 (4)	0.000
N2A	0.0123 (6)	0.0303 (7)	0.0130 (6)	0.000	0.0045 (5)	0.000
N5A	0.0117 (6)	0.0342 (8)	0.0178 (7)	0.000	0.0018 (5)	0.000
N6A	0.0101 (7)	0.0663 (12)	0.0164 (7)	0.000	0.0013 (5)	0.000
N7A	0.0109 (6)	0.0326 (8)	0.0106 (6)	0.000	0.0005 (5)	0.000
C3A	0.0107 (7)	0.0246 (8)	0.0136 (7)	0.000	0.0017 (5)	0.000
C4A	0.0117 (7)	0.0286 (8)	0.0170 (8)	0.000	0.0019 (6)	0.000
C8A	0.0159 (7)	0.0315 (9)	0.0145 (7)	0.000	0.0025 (6)	0.000
O1B	0.0137 (6)	0.0467 (8)	0.0141 (6)	0.000	0.0026 (4)	0.000
O9B	0.0177 (6)	0.0349 (7)	0.0143 (6)	0.000	−0.0027 (4)	0.000
N2B	0.0126 (6)	0.0404 (9)	0.0159 (7)	0.000	0.0017 (5)	0.000
N5B	0.0150 (6)	0.0361 (8)	0.0127 (6)	0.000	0.0006 (5)	0.000
N6B	0.0116 (7)	0.0540 (10)	0.0117 (6)	0.000	−0.0012 (5)	0.000
N7B	0.0113 (6)	0.0338 (8)	0.0109 (7)	0.000	0.0005 (5)	0.000
C3B	0.0136 (7)	0.0257 (8)	0.0133 (7)	0.000	0.0008 (6)	0.000
C4B	0.0128 (7)	0.0238 (8)	0.0142 (7)	0.000	0.0004 (5)	0.000
C8B	0.0137 (7)	0.0294 (9)	0.0169 (8)	0.000	−0.0010 (6)	0.000

### N-(4-Amino-1,2,5-oxadiazol-3-yl)formamide Geometric parameters (Å, º)

**Table:**

O1A—N2A	1.3888 (16)	O1B—N2B	1.3945 (17)
O1A—N5A	1.4082 (17)	O1B—N5B	1.4064 (17)
O9A—C8A	1.219 (2)	O9B—C8B	1.218 (2)
N2A—C3A	1.294 (2)	N2B—C3B	1.297 (2)
N5A—C4A	1.306 (2)	N5B—C4B	1.307 (2)
N6A—H6A	0.88 (3)	N6B—H6C	0.88 (2)
N6A—H6B	0.83 (3)	N6B—H6D	0.85 (3)
N6A—C4A	1.351 (2)	N6B—C4B	1.341 (2)
N7A—H7A	0.87 (3)	N7B—H7B	0.91 (3)
N7A—C3A	1.385 (2)	N7B—C3B	1.389 (2)
N7A—C8A	1.355 (2)	N7B—C8B	1.360 (2)
C3A—C4A	1.446 (2)	C3B—C4B	1.444 (2)
C8A—H8A	0.9500	C8B—H8B	0.9500
			
O1A···N7B^i^	3.0352 (18)	H6B···O9B^ii^	2.26 (3)
O1B···C8B^ii^	3.1671 (5)	N2A···N6B^x^	3.039 (2)
O9A···N6B^iii^	2.812 (2)	N2B···C8B^v^	3.1849 (5)
O9A···N2A	2.7947 (18)	N6A···N7A	3.054 (2)
N6A···O9A^iv^	2.9206 (19)	N6B···N7B	3.014 (2)
O9B···N7A^v^	2.8032 (18)	N2A···H6D^iii^	2.20 (3)
O9B···N6A^vi^	3.074 (2)	H6B···N2B^ii^	2.69 (3)
O9B···N2B	2.7448 (19)	N5A···H7B^vii^	2.32 (3)
O1A···H7B^vii^	2.24 (3)	N5A···H6C^vii^	2.31 (2)
O1B···H8A^viii^	2.3189	H8B···N5B^xi^	2.55
H6D···O9A^ix^	2.31 (3)	N6B···H7B	2.72 (3)
O9A···H6C^iii^	2.66 (2)	C8A···H6A^xii^	2.74 (3)
H6A···O9A^iv^	2.06 (3)	H6B···H7A	2.35 (3)
H6D···O9A^iii^	2.31 (3)	H6C···H7B	2.24 (4)
O9B···H7A^vi^	1.94 (3)		
			
N2A—O1A—N5A	110.56 (11)	N2B—O1B—N5B	111.41 (11)
C3A—N2A—O1A	105.44 (12)	C3B—N2B—O1B	104.56 (13)
C4A—N5A—O1A	105.71 (12)	C4B—N5B—O1B	104.97 (13)
H6A—N6A—H6B	120 (2)	H6C—N6B—H6D	118 (2)
C4A—N6A—H6A	119.8 (17)	C4B—N6B—H6C	121.3 (15)
C4A—N6A—H6B	119.8 (17)	C4B—N6B—H6D	120.5 (18)
C3A—N7A—H7A	119.6 (16)	C3B—N7B—H7B	116.9 (15)
C8A—N7A—H7A	114.5 (16)	C8B—N7B—H7B	118.0 (15)
C8A—N7A—C3A	125.88 (14)	C8B—N7B—C3B	125.04 (14)
N2A—C3A—N7A	125.07 (14)	N2B—C3B—N7B	125.41 (14)
N2A—C3A—C4A	110.23 (13)	N2B—C3B—C4B	110.49 (14)
N7A—C3A—C4A	124.71 (14)	N7B—C3B—C4B	124.10 (14)
N5A—C4A—N6A	124.68 (15)	N5B—C4B—N6B	125.28 (15)
N5A—C4A—C3A	108.07 (14)	N5B—C4B—C3B	108.56 (14)
N6A—C4A—C3A	127.25 (15)	N6B—C4B—C3B	126.16 (15)
O9A—C8A—N7A	125.23 (15)	O9B—C8B—N7B	123.42 (15)
O9A—C8A—H8A	117.4	O9B—C8B—H8B	118.3
N7A—C8A—H8A	117.4	N7B—C8B—H8B	118.3
			
O1A—N2A—C3A—N7A	180.000 (1)	O1B—N2B—C3B—N7B	180.000 (1)
O1A—N2A—C3A—C4A	0.0	O1B—N2B—C3B—C4B	0.000 (1)
O1A—N5A—C4A—N6A	180.000 (1)	O1B—N5B—C4B—N6B	180.000 (1)
O1A—N5A—C4A—C3A	0.0	O1B—N5B—C4B—C3B	0.000 (1)
N2A—O1A—N5A—C4A	0.000 (1)	N2B—O1B—N5B—C4B	0.000 (1)
N2A—C3A—C4A—N5A	0.0	N2B—C3B—C4B—N5B	0.000 (1)
N2A—C3A—C4A—N6A	180.000 (1)	N2B—C3B—C4B—N6B	180.000 (1)
N5A—O1A—N2A—C3A	0.000 (1)	N5B—O1B—N2B—C3B	0.000 (1)
N7A—C3A—C4A—N5A	180.0	N7B—C3B—C4B—N5B	180.000 (1)
N7A—C3A—C4A—N6A	0.000 (1)	N7B—C3B—C4B—N6B	0.000 (1)
C3A—N7A—C8A—O9A	0.000 (1)	C3B—N7B—C8B—O9B	0.000 (1)
C8A—N7A—C3A—N2A	0.000 (1)	C8B—N7B—C3B—N2B	0.000 (1)
C8A—N7A—C3A—C4A	180.000 (1)	C8B—N7B—C3B—C4B	180.000 (1)

Symmetry codes: (i) −*x*+1, *y*+1/2, −*z*; (ii) −*x*+1, *y*+1/2, −*z*+1; (iii) −*x*, −*y*+1, −*z*; (iv) *x*+1, *y*, *z*; (v) −*x*+1, *y*−1/2, −*z*+1; (vi) −*x*+1, −*y*+1, −*z*+1; (vii) −*x*+1, −*y*+1, −*z*; (viii) −*x*, −*y*+1, −*z*+1; (ix) −*x*, *y*−1/2, −*z*; (x) −*x*, *y*+1/2, −*z*; (xi) *x*+1, −*y*+1/2, *z*; (xii) *x*−1, *y*, *z*.

### N-(4-Amino-1,2,5-oxadiazol-3-yl)formamide Hydrogen-bond geometry (Å, º)

**Table:**

*D*—H···*A*	*D*—H	H···*A*	*D*···*A*	*D*—H···*A*
N6*A*—H6*A*···O9*A*^iv^	0.88 (3)	2.05 (3)	2.9207 (19)	172 (2)
N6*A*—H6*B*···O9*B*^vi^	0.83 (3)	2.26 (3)	3.074 (2)	164 (2)
N7*A*—H7*A*···O9*B*^ii^	0.87 (3)	1.93 (3)	2.8033 (18)	177 (2)
N7*A*—H7*A*···O9*B*^vi^	0.87 (3)	1.93 (3)	2.8033 (18)	177 (2)
N6*B*—H6*C*···N5*A*^vii^	0.88 (2)	2.31 (2)	3.189 (2)	175 (2)
N6*B*—H6*D*···O9*A*^iii^	0.85 (3)	2.31 (3)	2.8121 (19)	118 (2)
N6*B*—H6*D*···N2*A*^iii^	0.85 (3)	2.20 (3)	3.0387 (19)	166 (3)
N7*B*—H7*B*···O1*A*^vii^	0.91 (3)	2.24 (3)	3.0354 (17)	145 (2)
N7*B*—H7*B*···N5*A*^vii^	0.91 (3)	2.32 (3)	3.2302 (19)	179 (2)
C8*A*—H8*A*···O1*B*^viii^	0.95	2.32	3.266 (2)	175
C8*B*—H8*B*···N5*B*^xi^	0.95	2.55	3.489 (2)	170

Symmetry codes: (ii) −*x*+1, *y*+1/2, −*z*+1; (iii) −*x*, −*y*+1, −*z*; (iv) *x*+1, *y*, *z*; (vi) −*x*+1, −*y*+1, −*z*+1; (vii) −*x*+1, −*y*+1, −*z*; (viii) −*x*, −*y*+1, −*z*+1; (xi) *x*+1, −*y*+1/2, *z*.
